# Research trends and hot spots in obesity-induced pain: A bibliometric analysis of the last 20 years

**DOI:** 10.1016/j.ibneur.2025.02.001

**Published:** 2025-02-10

**Authors:** Lei Gao, Yazhou Wen, Kunlin Guo, Renqi Li, Mao Mao, Shanwu Feng, Xian Wang

**Affiliations:** Department of Anesthesiology, Women’s Hospital of Nanjing Medical University, Nanjing Women and Children’s Healthcare Hospital, Nanjing, Jiangsu, China

**Keywords:** Obesity, Pain, Bibliometric analysis, Research trends, VOSviewer, CiteSpace

## Abstract

**Background:**

Obesity can directly lead to allodynia, increase the incidence of chronic pain, and aggravate existing pain. However, the mechanisms underlying obesity-related or obesity-induced pain are still not understood. Herein, we performed a comprehensive bibliometric analysis of obesity-related or obesity-induced pain, aiming to analyze the current trends and hot spots as well as explore the underlying mechanisms.

**Methods:**

We searched reviews and articles on obesity-related or obesity-induced pain from 2005 to 2024 via the Web of Science Core Collection (WoSCC) database. We subsequently conducted bibliometric analysis employing WPS Office, a web-based bibliometric analysis platform (https://bibliometric.com), VOSviewer, Pajek, and CiteSpace.

**Results:**

In total, 347 papers were identified for bibliometric analysis. The country, institution, and journal with the greatest influence were the USA, Albert Einstein College of Medicine, and Headache, respectively. Dr. Lipton RB and Dr. Karppinen J were the top 2 influential authors on the basis of their significant number of publications and citations. The keywords for the latest burst were "inflammation," "risk," "neuropathic pain," "gene-related peptide," "knee osteoarthritis," and "validation." Notably, the article titled "The association between chronic obesity and pain" by Okifuji A received the highest number of citations as well as the strongest citation burst. He and colleagues noted a significant correlation between obesity and pain in terms of clinical manifestations, but this connection is indirect and is modulated by certain biomechanical and structural alterations linked to obesity, inflammatory agents, mood disorders, sleep disturbances, and lifestyles.

**Conclusion:**

There has been a notable surge in the number of articles published in the last two decades. The investigation into neuroendocrine and neuroimmune mechanisms underlying obesity-related or obesity-induced pain is expected to be a hot spot in the coming years. A potential strategy for treating chronic obesity and pain should pay attention to particular endocrine regulators, inflammatory cytokines, or immune cells that serve as central elements or crucial signaling pathways within this regulatory system.

## 1 Introduction

Obesity and pain are two major health problems that significantly endanger the lives and health of the public (Wright et al., 2010, Stone and Broderick, 2012). Obesity, as per the World Health Organization's definition, refers to the abnormal or excessive buildup of fat that poses a health risk. In addition, pain is a debilitating symptom that significantly affects physical and emotional functioning, decreases the capacity to work, and reduces quality of life (Taylor et al., 2016, Walker et al., 2014). Notably, obesity is related to various health issues, including chronic pain (Guh et al. 2009). Obesity can directly lead to mechanical and thermal allodynia in mice fed a Western diet (Elshareif et al. 2023), increase the incidence of chronic pain, such as low back pain, migraine, and fibromyalgia (Sarzi-Puttini et al. 2020), or aggravate existing pain, including neuropathic, inflammatory, postoperative, and visceral pain (Zhang et al. 2023b).

An investigation of middle-aged individuals in the USA revealed that an increase in body mass index (BMI) is responsible for approximately 20 % of the increase in mild/moderate or nonlimiting pain and 32 % of the increase in severe or limiting pain in women, whereas the corresponding percentages is 10 % and 19 % for men (Stokes et al. 2020). Similar results are observed in adolescents and children (Paulis et al., 2014, Hainsworth et al., 2021). By contrast, weight loss has been demonstrated to dramatically reduce pain in both clinical trials and observational studies (Narouze and Souzdalnitski, 2015, Dong et al., 2021). The coexistence of obesity and pain can exacerbate a patient's functional capacity and decrease their life quality to a greater extent than either condition alone. Notably, there has been a substantial increase in the occurrence of obesity in recent years (Li et al. 2022). Therefore, such obesity-related or obesity-induced pain phenomena have attracted our attention.

The mechanism underlying obesity-induced pain is complex and still not fully understood. Excess weight may partially explain the increased incidence of weight-bearing joints, such as hip and knee pain, but it does not explain the increased incidence of hand/finger arthritis pain or migraine. One meta-analysis proposed that body fat, rather than body weight, was a better risk indicator for musculoskeletal pain (Hsu et al. 2019). Further, genes, lifestyle, or metabolism affect the pain threshold caused by obesity. C57BL/6CR and C57BL/6NJ mice fed the same high-fat diet developed significant mechanical hypersensitivity. By contrast, C57BL/6 J mice did not experience obesity-induced pain because they were more physically active than the other two substrains. C57BL/6 J mice have a mutation in nicotinamide nucleotide transhydrogenase, a mitochondrial protein that has a significant effect on insulin secretion and glucose metabolism. Disorder of the gut-brain axis is another recognized mechanism underlying pain regulation. Butyrate, an intestinal microorganism-derived metabolite that is highly enriched in obese individuals, affects the lipid metabolism and calcium homeostasis of dorsal root ganglion neurons and peripheral nerve immune cells such as macrophages and Schwann cells, leading to peripheral neuropathy and pain (Bonomo et al. 2020).

Furthermore, as an endocrine organ, adipose tissue secretes a variety of neuromodulators, such as leptin, galanin, and ghrelin, which promote appetite, metabolism, and pain perception, possibly via interactions with certain neurotransmitters or pathways, such as N-methyl-D-aspartic acid receptor (NMDA), cannabinoid 1 receptor, or the opioid system, to promote central sensitization (Zhang et al. 2023a). Adipose cells recruit macrophages and release cytokines, such as IL-6 and TNF-α, which present a pronociceptive effect (Surmi and Hasty, 2008). Obesity-related dyslipidemia can reduce the blood supply and increase oxidative stress in large myelinated nerves and small sensory nerve fibers, leading to peripheral neuropathy and pain (Xu et al. 2014).

However, bibliometric analysis of obesity-related or obesity-induced pain phenomena comprehensively is lacking. Therefore, we used the Web of Science Core Collection (WoSCC) database to search for available publications of obesity-related or obesity-induced pain in the past two decades. Then, WPS Office, a web-based bibliometric analysis platform (https://bibliometric.com), VOSviewer, Pajek, and CiteSpace software are employed to identify trends and hot spots of obesity-related or obesity-induced pain via visualization (Wormell, 2003, van Eck and Waltman, 2010, Chen, 2006, Zyoud and Fuchs-Hanusch, 2017). We hope this study will provide promising insights into this academic framework to benefit future study.

## 2 Methods

### 2.1 Literature study

We searched the WoSCC database for articles and reviews that had recently been published regarding obesity-related or obesity-induced pain. Publications, not articles or reviews, such as meeting abstracts, letters, or editorials, were excluded. The data were obtained on December 31, 2024. The following terms were used in the search: TI = (pain∗ OR headache∗ OR migraine∗ OR head-ache∗ OR "head ache∗" OR cephalalgi∗ OR "abdominal ache∗" OR fibromyalg∗ OR "tummy ache∗" OR "stomach ache∗" OR "belly ache∗" OR ear-ache∗ OR earache∗ OR tooth-ache∗ OR toothache∗ OR odontalgi∗ OR neuralgi∗ OR cervicodyn∗ OR analg∗ OR nocicept∗ OR hyperalg∗ OR hypoalg∗ OR radiculalg∗ OR colic OR arthralg∗ OR causalg∗ OR maldyn∗ OR eudyn∗ OR ophthalmodyn∗ OR cephalalg∗ OR dysmenorrh∗ OR sciatic∗ OR otalg∗ OR brachialg∗) AND TI = (obesity OR overweight).

### 2.2 Data collection

We extracted bibliometric data on obesity-related or obesity-induced pain, including title, publication year, citation count, nation or area, institution, author, journal, keywords, and references, from the WoSCC database. The documents and references were exported in a plain text file format named "download_* ** ."

### 2.3 Data analysis and visualization

We analyzed the yearly publication via the WPS Office. The cooperation information among different nations was analyzed and presented via a web-based bibliometric analysis platform (https://bibliometric.com). With the aid of VOSviewer 1.6.19 and Pajek32 5.18, the co-authorship analysis of writers, countries, and institutions was carried out. The keywords and co-cited references were examined using CiteSpace 6.2.R4.

## 3 Results

### 3.1 Publications inclusion

**Fig. 1:**
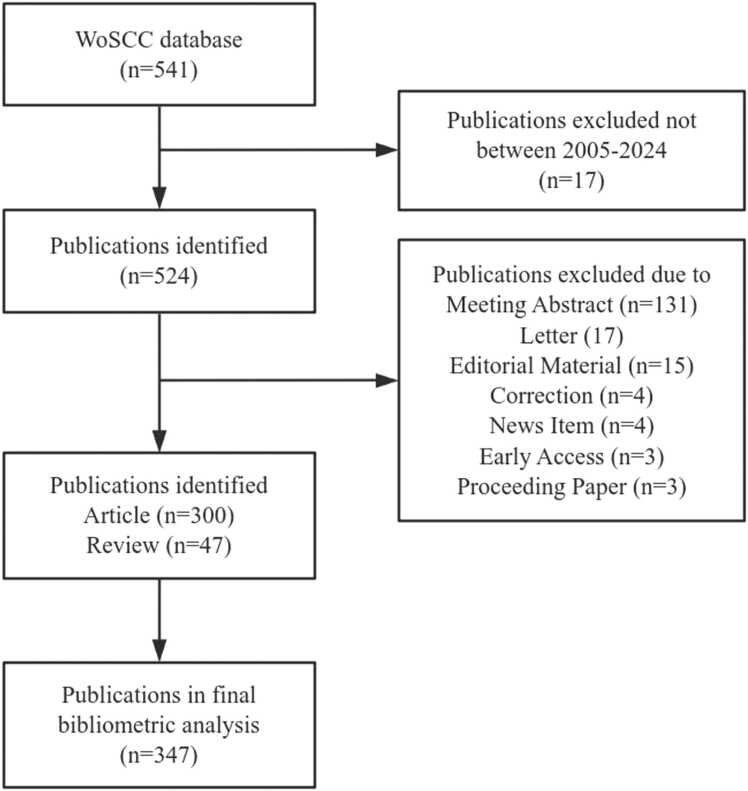
Flow chart of study inclusion.

After the WoSCC database was searched, a total of 541 publications related to obesity-related or obesity-induced pain were screened. After further excluding publications not meeting the inclusion criteria, we ultimately identified 347 publications for bibliometric analysis. Fig. 1 displayed a comprehensive flowchart illustrating the screening procedure.

### 3.2 Yearly publications and citations

**Fig. 2:**
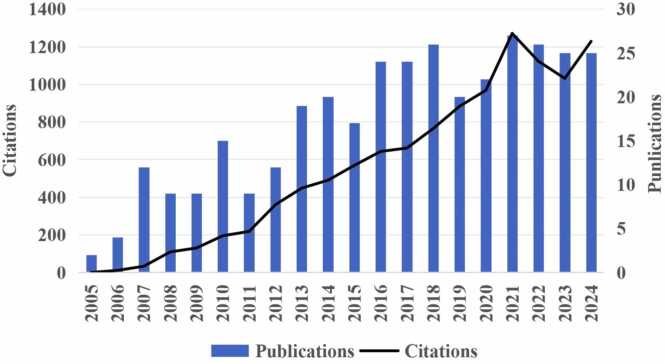
Annual publications and citations for obesity-induced pain studies.

From Jan 1, 2005, to Dec 31, 2024, a total of 347 publications were included in the final bibliometric analysis. The combined number of citations was 11,160, with an average of 32.16 citations per publication. The h-index for these publications was 57. Fig. 2 illustrated a progressive increase in the annual number of citations from 2005 to 2024, reaching its peak in 2021 with 1270 citations.

### 3.3 Role and impact of countries and institutions

**Fig. 3:**
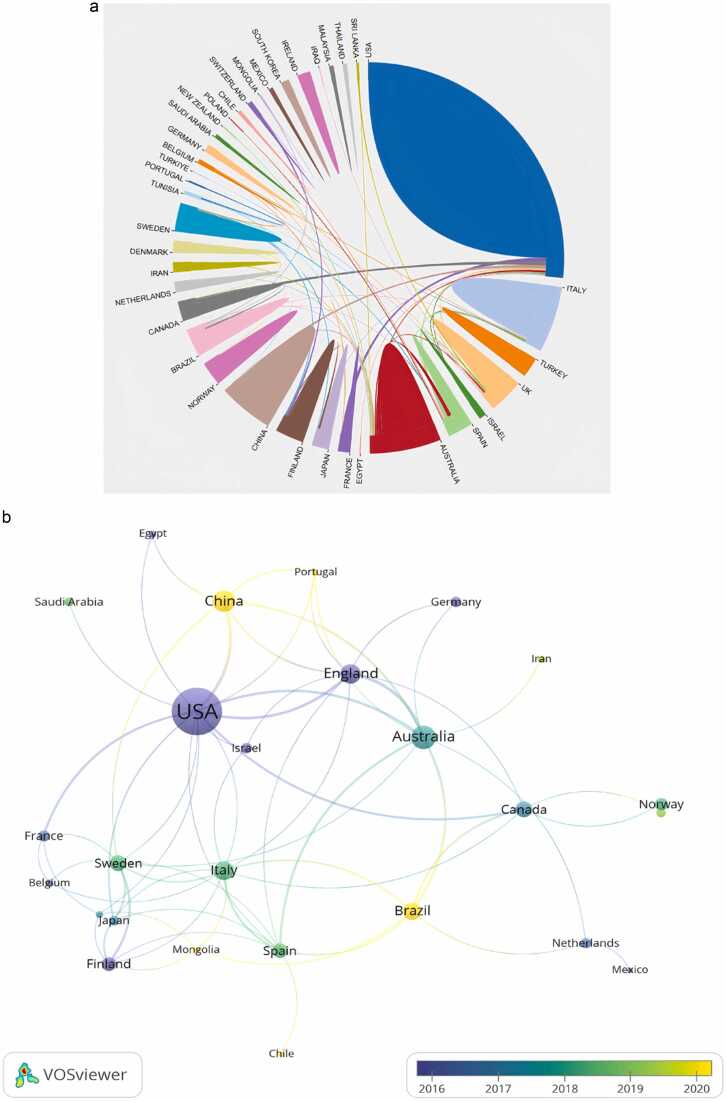
**Role and impact of nations and institutions in obesity and pain studies**. (A) Global distribution and collaboration across nations for obesity and pain research. (B) Map of national cooperation for pain and obesity research (threshold = 2). The color of the nodes and the thickness of the lines represent the mean year of publication and the frequency of international collaboration, respectively.

According to Fig. 3A, 39 nations or areas contributed to the 347 publications. The USA had the highest number of publications among all countries. Table 1 showed that the USA ranked first with 144 publications, followed by Australia (n = 36), China (n = 30), and England (n = 24). However, as Fig. 3B illustrated, nations such as China and Brazil are more active in recent years. With respect to collaboration among countries, cooperation between England and Australia ranked first (n = 7), followed by that between the USA and China (n = 6).

**Table 1:** The 10 most influential nations or regions in obesity-induced pain studies.

Rank	Country/Region	Publications	Citations	Total Link Strength
1	USA	144	5914	35
2	Australia	36	1279	27
3	China	30	646	14
4	England	24	890	21
5	Italy	22	593	15
6	Brazil	19	297	12
7	Sweden	16	281	14
8	Canada	16	359	11
9	Spain	14	458	15
10	Turkey	13	153	0

**Fig. 4:**
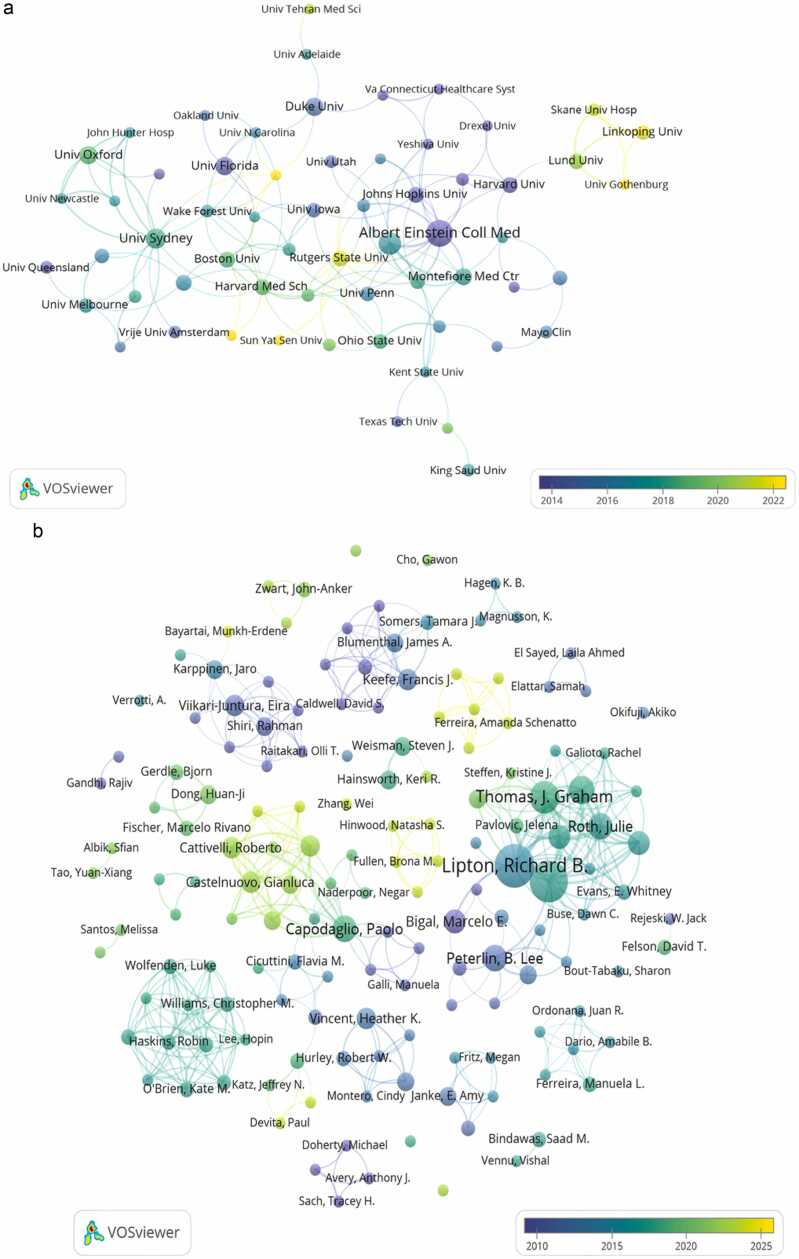
**Institutions and authors coauthorship network maps**. (A) A cooperative network map involving 81 universities with three or more publications. The color of the nodes and the thickness of the lines represent the mean year of publication and the frequency of collaboration among institutions, respectively. (B) A cooperative network map created by authors with collaborative relationships. The color of the nodes and the thickness of the lines represent the mean year of publication and the frequency of author collaboration, respectively.

A total of 637 institutions were identified as contributors to obesity-related or obesity-induced pain research. Of these, 81 institutions published three or more papers. Fig. 4A indicated that among the 81 institutions, 61 engaged in collaboration with one another. Table 2 presented details regarding the leading 10 schools, of which 6 were universities in the USA. Albert Einstein College of Medicine stood out as the institution with the highest number of papers (n = 17). Further, Brown University had the highest link strength, with a count of 27, followed by Albert Einstein College of Medicine with 26 and the University of Sydney with 25. The strength of a link indicated the strength of a collaboration.

**Table 2:** The 10 most influential institutions in obesity-induced pain studies.

Rank	Institution	Country	Publications	Citations	Total Link Strength
1	Albert Einstein College of Medicine	USA	17	1248	26
2	Brown University	USA	12	287	27
3	University of Sydney	Australia	10	348	25
4	University of Oxford	England	8	138	10
5	Duke University	USA	8	211	4
6	University of Florida	USA	8	241	4
7	Montefiore Medical Center	USA	7	89	16
8	University of Oslo	Norway	7	136	6
9	The Finnish Institute of Occupational Health	Finland	7	848	5
10	Harvard Medical School	USA	6	191	15

### 3.4 Role and impact of authors

A total of 1783 authors were identified as contributors to obesity-related or obesity-induced pain research, with 156 having 2 or more publications. Table 3 displayed the top 10 writers who have received the highest number of citations and publications. Dr. Lipton RB., Karppinen J., and Viikari-Juntura E. were among the most influential authors on the basis of their significant number of citations. Dr. Lipton RB, Bond DS, and Thomas JG were considered the most prominent authors on the basis of their extensive publishing records.

**Table 3:** The 10 most influential authors in obesity-induced pain studies.

Rank	Author	Citations	Rank	Author	Articles
1	Lipton RB	927	1	Lipton RB	16
2	Karppinen J	808	2	Bond DS	15
3	Viikari-Juntura E	807	3	Thomas JG	10
4	Shiri R	741	4	Pavlovic, JM	8
5	Solovieva S	642	5	Peterlin BL	7
6	Bigal, ME	641	6	Capodaglio P	7
7	Leino-Arjas P	619	7	Roth J	7
8	Okifuji A	525	8	Bigal, ME	6
9	Peterlin BL	520	9	Rathier L	6
10	Bond DS	411	10	O'Leary, KC	6

The coauthorship network map in Fig. 4B displayed the collaborative links among the top 156 authors. Additionally, there were several research groups in this field, including the group led by Dr. Capodaglio P. and the group led by Lipton RB. Dr. Lipton RB, Bond DS, Thomas JG, and their colleagues have become the most prominent research group.

### 3.5 Journal analysis

In total, 197 journals published articles on obesity-related or obesity-induced pain. Table 4 displayed the top 10 journals. The impact factor (IF) for 2024 varied between 2.4 and 5.9. Among them, Headache has the most extensive collection of articles (n = 10, IF 5.4), followed by the European Journal of Pain (n = 8, IF 3.5) and Obesity (n = 8, IF 4.2).

**Table 4:** The 10 most influential journals in obesity-induced pain studies.

Rank	Journal	Documents	Quartile in category	IF (2024)
1	Headache	10	Q1	5.4
2	European Journal of Pain	8	Q2	3.5
3	Obesity	8	Q1	4.2
4	Current Pain and Headache Reports	7	Q2	3.2
5	BMC Public Health	7	Q1	3.5
6	Journal of Pain	6	Q1	4.0
7	BMJ Open	6	Q1	2.4
8	Frontiers In Endocrinology	6	Q2	3.9
9	Pain	5	Q1	5.9
10	Clinical Journal of Pain	5	Q2	2.6

Notes: Q1: JCR divides the journals into 176 different discipline categories, each of which is classified into Q1–4 according to the impact factor of the journal in the current year. The journal with the top 25 % (including 25 %) impact factors is Q1. Journals with impact factors ranging from 25 % to 50 %, 50 %–75 %, and after 75 % are Q2, Q3, and Q4, respectively.

### 3.6 Keyword analysis

**Fig. 5:**
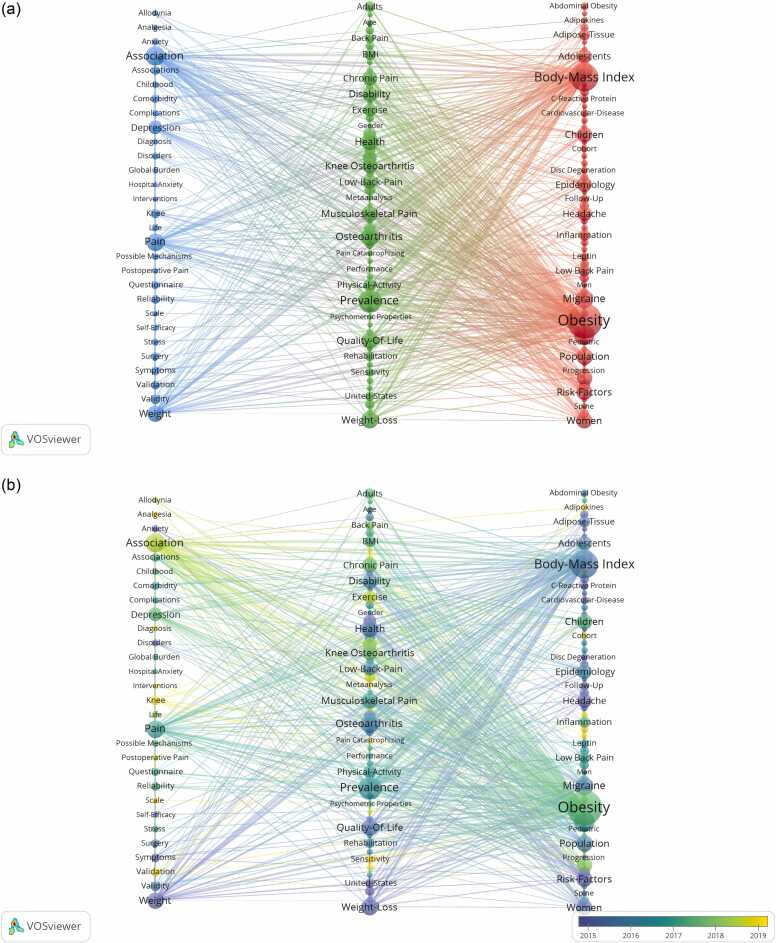
**VOSviewer network diagram for keyword analysis visualization**. (A) Analyses of clusters based on the 141 most frequently occurring terms in the fields of obesity and pain research. (B) Keyword timeline view. The size and color of the nodes indicate the number of citations and the mean year published. A shorter distance between nodes leads to a higher degree of keyword co-occurrence.

We detected 1508 keywords related to obesity-related or obesity-induced pain research. Table 5 showed the top 20 keywords ranked by their occurrence and the corresponding total link strength for each term. Next, we created a visual representation of the network of terms that occur together, using a total of 141 keywords that appear at least 5 times. Fig. 5A showed that keywords within the same cluster are represented by nodes of similar color. In the blue cluster, "association," "pain," "weight," "depression," "knee," and "stress" were highly enriched. In the green cluster, the significantly enriched nodes were "prevalence," "quality-of-life," "weight-loss," and "health". In the red cluster, "body mass index," "obesity," "risk factors," "epidemiology," and "headache" attracted considerable attention. Fig. 5B displayed a graphical representation of how keywords have changed over time, which helps to identify areas of intense activity in the field. The colors of the nodes represented the mean year in which the keywords appeared.

**Table 5:** The 20 most important keywords in obesity-induced pain studies.

Rank	Keyword	Occurrences	Total Link Strength	Rank	Keyword	Occurrences	Total Link Strength
1	Obesity	182	1301	11	Population	40	322
2	Body-mass Index	114	838	12	Weight	39	299
3	Prevalence	77	554	13	Musculoskeletal Pain	38	281
4	Overweight	62	508	14	Women	38	272
5	Association	56	437	15	Risk-factors	37	278
6	Osteoarthritis	48	370	16	Disability	35	251
7	Pain	45	323	17	Children	34	258
8	Health	43	295	18	Knee Osteoarthritis	33	258
9	Weight-loss	42	319	19	Chronic Pain	32	268
10	Quality-of-life	41	317	20	Exercise	30	198

**Fig. 6:**
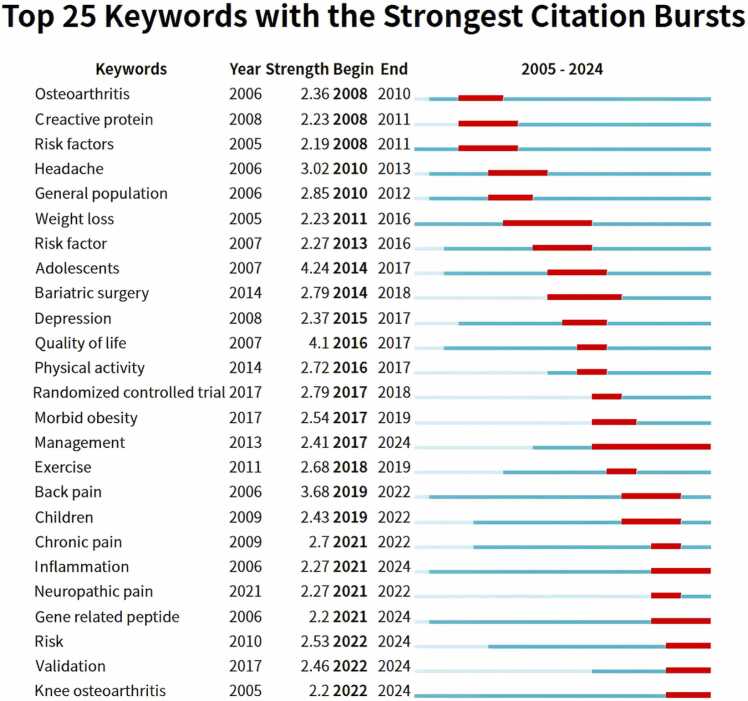
The 25 most-cited research keywords related to obesity-induced pain bursts.

Fig. 6 showed the 25 most frequently cited keywords with a sudden increase in citation frequency, reflecting study trends and hot spots at various times. The five keywords that cause the most severe outbreaks were "adolescents," "quality of life," "back pain," "headache," and "general population." The keywords exhibiting the most extended burst duration were "management," "weight loss," and "bariatric surgery." Moreover, the recent outbreak keywords included "inflammation," "risk," "neuropathic pain," "gene-related peptide," "knee osteoarthritis," and "validation." These results provided insights into the current hot spots of research on obesity and pain.

### 3.7 Cocited reference analysis

A co-citation connection was formed when a third publication cites two references. The 10 most cocited articles on obesity-related or obesity-induced pain were shown in Table 6. The article titled "The association between chronic obesity and pain" by Okifuji A, published in the Journal of Pain Research in 2015, received the highest number of citations (n = 32) (Okifuji and Hare, 2015). Following this, "The association between obesity and low back pain: a meta-analysis" by Shiri R, published in the American Journal of Epidemiology in 2009, received 31 citations, ranking second (Shiri et al. 2010).

**Table 6:** The 10 most co-cited publications in obesity-induced pain studies.

Rank	Title	Journal (IF)	First Author	Publication Time	Country	Co-Citation	Quartile in Category
1	The association between chronic pain and obesity	Journal of Pain Research (2.7)	Okifuji A	Jul,2015	USA	32	Q2
2	The association between obesity and low back pain: a meta-analysis	American Journal of Epidemiology (5.0)	Shiri R	Dec,2009	Finland	31	Q1
3	Overview of the relationship between pain and obesity: What do we know? Where do we go next?	Journal of Rehabilitation Research and Development (1.3)	Janke EA	Jan, 2007	USA	30	Q4
4	Obesity and pain are associated in the United States	Obesity (4.2)	Stone AA	Jan,2012	USA	30	Q1
5	Body weight and low back pain. A systematic literature review of 56 journal articles reporting on 65 epidemiologic studies	Spine (2.6)	LeBeouf C	Jan,2000	Denmark	24	Q1
6	The more pain I have, the more I want to eat": obesity in the context of chronic pain	Obesity (4.2)	Janke EA	Feb,2012	USA	21	Q1
7	Chronic pain, overweight, and obesity: findings from a community-based twin registry	Journal of Pain (4.0)	Wright LJ	Jul,2010	USA	19	Q1
8	Obesity and chronic pain: systematic review of prevalence and implications for pain practice	Regional Anesthesia and Pain Medicine (5.1)	Narouze S	Mar,2015	USA	19	Q1
9	Comorbidity of obesity and pain in a general population: results from the Southern Pain Prevalence Study	Journal of Pain (4.0)	Holli CH	Mar, 2007	USA	18	Q1
10	Mechanisms of association between obesity and chronic pain in the elderly	Pain (5.9)	Ray L	Jan,2011	USA	18	Q1

**Fig. 7:**
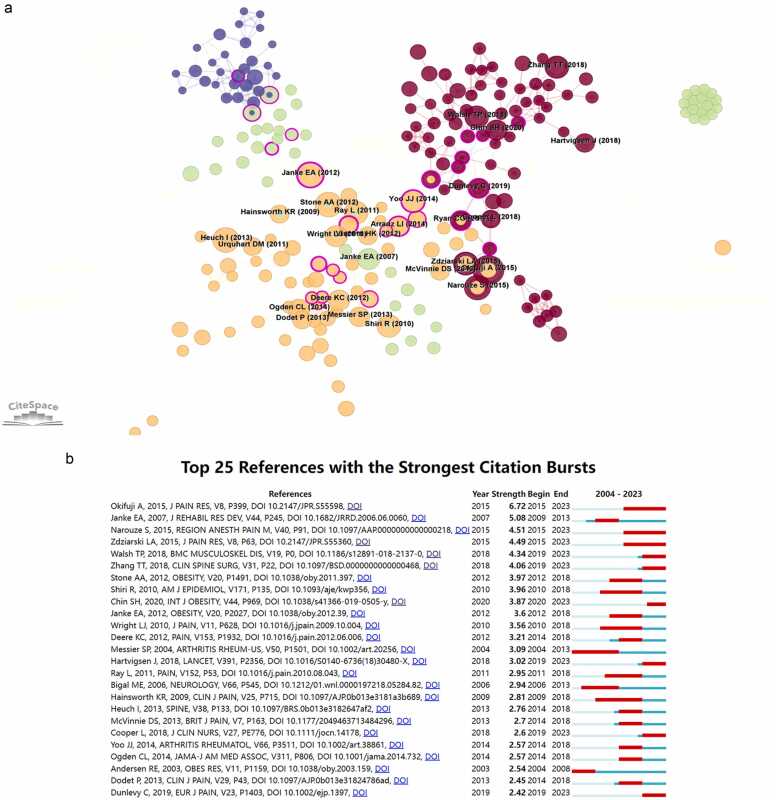
**Analyzing references in the field of obesity-induced pain via CiteSpace**. (A) The depicted network represents the interconnected references pertaining to research on obesity and pain. With CiteSpace, we specify the following parameters: timespan: 2005–2024 (slice length = 5), node type (reference), selection criterion: g index (k = 25), and pruning: Pathfinder. A network consisting of 261 nodes and 537 links was constructed on the basis of the given parameters. (B) The 25 most-cited references on the topic of obesity and pain bursts.

Fig. 7A displayed the visual representation of the network of cited references, which was generated via CiteSpace software. Nodes were colored in accordance with the publication date of the reference. A node's centrality was denoted by a purple circle surrounding it, which signified its significance within the field. The reference citation frequency was denoted by the node size. Fig. 7B displayed the 25 most important references exhibiting the most pronounced citation bursts, showing that these articles were frequently cited during a condensed time. The article "Okifuji A, 2015, J PAIN RES, V8, P399 (Okifuji and Hare, 2015)" achieved the maximum burst value of 6.72; "Janke EA, 2007, J REHABIL RES DEV, V44, P245 (Janke, Collins, and Kozak, 2007)" came in second place with a burst value of 5.08; and "Narouze S, 2015, REGION ANESTH PAIN M, V40, P91 (Narouze and Souzdalnitski, 2015)" achieved a burst value of 4.51, ranking third. These articles offered insights into the hot spots associated with obesity-related or obesity-induced pain phenomena.

## 4 Discussion

This study performed methodological research with respect to obesity-related or obesity-induced pain over the past two decades. As presented, there was a progressive increase in the annual number of publications and citations from 2005 to 2024. The USA had made a significant contribution in terms of publications, citations, international cooperation, and research institutions. Further, Albert Einstein College of Medicine had performed multiple studies on the effect of obesity on pain. The authors, Dr. Lipton RB and Dr. Karppinen J, were the 2 most influential authors on the basis of their significant numbers of publications and citations, respectively. Further, the keywords for the latest burst were "inflammation," "risk," "neuropathic pain," "gene-related peptide," "knee osteoarthritis," and "validation." Notably, the article titled "The association between chronic obesity and pain" by Okifuji A, published in the Journal of Pain Research in 2015, received the highest number of citations as well as the strongest citation burst.

Dr. Lipton RB published 16 articles, representing 4.7 % of the overall articles, and he had the highest cumulative number of citations. In one of his highly referenced works (McCarthy et al. 2009), the authors suggested that chronic pain is observed in as many as 52 % of elderly individuals. Further, chronic pain is twice as likely or more prevalent among obese individuals than among normal-weight individuals. In another article (Ray et al. 2011), he suggested that central obesity predicted higher total pain index scores (OR=1.55; 95 % CI: 1.04--2.33) and nearly doubled the risk of chronic pain (OR=1.70; 95 % CI: 1.05--2.75) in elderly individuals. They reported that, as a metabolic syndrome component, central obesity presented the strongest independent association with pain.

Dr. Karppinen J. possessed the second-highest cumulative number of citations among the authors. In his most referenced publication, he examined the correlation between obesity and low back pain and reported that being overweight or obese increased the likelihood of experiencing low back pain (OR = 1.53, 95 % CI: 1.22, 1.92) (Shiri et al. 2010). Overweight and obesity are strongly linked to seeking medical treatment for low back pain. In addition, another article published in 2011 (Samartzis et al. 2011) indicated that being overweight was closely linked to the occurrence of disc degeneration in adolescents, leading to more severe low back pain and reduced physical and social capabilities. There was a strong correlation between a higher BMI and more severe disc degradation (OR = 14.19; 95 % CI: 1.44–140.40). These studies highlighted that being overweight *per se* was one contributor to certain pain conditions, such as low back pain.

Among the journals focused on obesity-related or obesity-induced pain research, the top 10 journals contributed 19.6 % (68 publications) of the overall publications. Headache published the most articles on obesity-related or obesity-induced pain research (2.9 %), followed by the European Journal of Pain (2.3 %), Obesity (2.3 %), and BMC Public Health (2.0 %). According to the 2023 edition of the Journal Citation Reports, the top 10 journals have IFs below 10. Among these journals, those with IFs between 2 % and 3 %, 3 % and 5 %, and 5 % and 10 % accounted for 30 %, 52 %, and 18 %, respectively. Accordingly, more high-quality publications are warranted in the future.

As demonstrated in Fig. 5, Fig. 6, the clustering of keywords and the keyword emergence map suggested that research on obesity-related or obesity-induced pain has shifted from describing phenomena to investigating the underlying mechanisms and potential solutions. Janke EA (Janke, Collins, and Kozak, 2007) proposed that the correlation between weight gain and various pain disorders is likely attributed to a mix of mechanical, structural, metabolic, and behavioral alterations. Moreover, Shiri R (Shiri et al. 2010) reported that the correlation between being overweight or obese and the occurrence of low back pain is more pronounced in women than in men, potentially attributed to hormone-related alterations linked to obesity and sensitivity to pain.

Importantly, weight loss and exercise are effective ways to relieve pain in obese patients. Wasser JG’s study (Wasser et al. 2017) revealed that obese people with lower back pain face a dilemma regarding whether to exercise due to pain interference, although the benefits of exercise in terms of weight loss, improved functioning, and quality of life have been well documented. To investigate this inquiry, he identified 16 programs on diverse exercise regimens, thereby evaluating the efficacy of treating symptoms of low back pain in obese individuals. Results revealed that yoga-Pilates exercise programs, resistance training, and aquatic exercise were the most effective methods for relieving pain in obese people. Additionally, he noted that the best way to maximize patients' quality of life is by minimizing pain-related movement anxiety and maintaining an exercise regimen. In Fig. 5B, the high frequency of "exercise," "weight loss," and "bariatric surgery" can also be good proof of this point.

Notably, the interrelationship between obesity and pain sensitivity is bidirectional (Yu et al. 2013). Pain disorders may also have contributed to the development of obesity. Patients experiencing pain complained that pain hindered their ability to lose weight (Choi et al. 2014). Preliminary evidence showed that patients with chronic obesity and pain had a lower success rate with treatment (Amy Janke and Kozak, 2012). Perhaps the most apparent cause-and-effect relationship is that pain increases the likelihood of obesity by diminishing exercise (Cunha et al. 2020). Further, individuals tend to consume a greater quantity of sugary food when experiencing pain, in addition to decreasing their level of physical activity (Amy Janke and Kozak, 2012), especially if they cannot control themselves, which could result in obesity (Darbor et al., 2016, Guimarães et al., 2021).

Of note, it is essential to understand the mechanisms of obesity-related or obesity-induced pain. One of the key neuroendocrine hormones is leptin, an adipokine secreted by adipose tissue. Leptin has been shown to influence both appetite and pain perception. High levels of leptin, often found in obese individuals, can lead to leptin resistance, which may disrupt the normal regulatory functions of the hormone (Zhang et al. 2023a). Leptin acts on the central nervous system, particularly in the hypothalamus, where it interacts with various neurotransmitter systems, including NMDA receptors, to modulate pain sensitivity (Wauman and Tavernier, 2011). Another important hormone is insulin, which is involved in glucose metabolism and also has effects on pain pathways. Insulin resistance, a common feature of obesity, can lead to increased levels of circulating insulin. Elevated insulin level is associated with enhanced pain perception, possibly through their actions on peripheral and central nociceptive pathways (Koonce and Bravman, 2013). Insulin can also influence the release of inflammatory cytokines, further exacerbating pain (Rojas et al. 2016).

The neuroimmune system is another critical pathway to explain how obesity influences pain. Obesity is characterized by a state of chronic low-grade inflammation, with increased levels of inflammatory cytokines such as IL-6 and TNF-α. These cytokines can directly sensitize nociceptive neurons, leading to increased pain perception (Le Thuc and García-Cáceres, 2024). For example, IL-6 can enhance the expression of ion channels and receptors involved in pain signaling, such as TRPV1 and Nav1.8, in dorsal root ganglion neurons. Additionally, obesity can lead to the activation of immune cells, such as macrophages and T-cells, which infiltrate adipose tissue and release pro-inflammatory cytokines. These cytokines can also affect the function of the gut-brain axis, leading to alterations in the gut microbiome and increased production of metabolites like butyrate. Butyrate has been shown to affect lipid metabolism and calcium homeostasis in dorsal root ganglion neurons and peripheral nerve immune cells, leading to peripheral neuropathy and pain (Agus, Clément, and Sokol, 2021).

Several clinical studies also support the role of neuroendocrine and neuroimmune mechanisms in obesity-related pain. Narouze found that weight loss interventions, such as bariatric surgery, led to significant reductions in pain scores, which correlated with improvements in insulin sensitivity and reductions in inflammatory markers. This suggests that addressing the neuroendocrine and neuroimmune dysregulation associated with obesity can effectively alleviate pain (Narouze and Souzdalnitski, 2015).

As the final part of this bibliometric analysis, we analyzed the visualization network of the cited references, and the work of Okifuji A, published in the Journal of Pain Research in 2015, attracted our attention (Okifuji and Hare, 2015). Okifuji A noted a significant correlation between obesity and pain in terms of clinical manifestations, but this connection was indirect and was modulated by various factors. These factors encompass biomechanical and structural alterations linked to obesity, inflammatory agents, mood disorders, sleep disturbances, and lifestyles. Additionally, there was extensive evidence indicating a significant occurrence of depression in those who suffer from obesity and pain. After adjusting for depression, the link between obesity and chronic pain was weakened, indicating that depression could impact the connection between obesity and pain. Similarly, other studies have shown that depression can impact alterations in various regions of the brain, particularly those related to the processing and perception of pain (Bär et al., 2007, Giesecke et al., 2005).

Although this is the first bibliometric analysis of the research trends in obesity-related or obesity-induced pain, it has several limitations. Firstly, bibliometric analysis provides few clues about the mechanism of obesity-related or obesity-induced pain. To address this problem, we discussed the underlying pronociception mechanism for obesity-related or obesity-induced pain on the basis of highly cited publications or those with a high frequency of keywords. Secondly, the publications obtained are solely from the WoSCC database, and only English publications are included. This could lead to inaccuracy in the prediction of research centers, as the literature from other databases is absent. Thirdly, while we strive to encompass all the search terms in our search formula, some synonyms may still be excluded, resulting in the omission of certain studies.

## 5 Conclusion

This study sheds light on obesity-related or obesity-induced pain research. There has been a notable surge in the quantity of articles published in the last two decades. The country, institution, author, and journal with the greatest influence are the USA, Albert Einstein College of Medicine, Lipton RB, and Headache, respectively. Notably, the article titled "The association between chronic obesity and pain" by Okifuji A received the highest number of citations as well as the strongest citation burst. The investigation into neuroendocrine and neuroimmune mechanisms underlying obesity-related or obesity-induced pain is expected to be a hot spot in the coming years. A potential strategy for treating chronic obesity and pain should pay attention to particular endocrine regulators, inflammatory cytokines, or immune cells that serve as central elements or crucial signaling pathways within this regulatory system.

## Ethical approval statement

Ethics approval statement is not applicable.

## Funding

This work is supported by grants from the 10.13039/501100001809National Natural Science Foundation of China (81971040, 81971045), the 10.13039/501100013058Jiangsu Provincial Key Research and Development Program (BE2021615), and the Nanjing Medical Science and Technology Development Fund (KBX23040).

## CRediT authorship contribution statement

**Wang Xian:** Writing – review & editing, Funding acquisition, Conceptualization. **Li Renqi:** Investigation, Software. **Mao Mao:** Validation, Project administration, Software. **Feng Shanwu:** Data curation, Resources, Supervision. **Gao Lei:** Writing – original draft, Data curation, Formal analysis. **Wen Yazhou:** Investigation, Methodology, Software. **Guo Kunlin:** Software, Investigation.

## Declaration of Competing Interest

The authors declare that they have no competing interests.

## Data Availability

The data that support the findings of this study are available from the corresponding author uponreasonable request.
